# Outbreak of *Vibrio cholerae* Serogroup O1, Serotype Ogawa, Biotype El Tor Strain — La Huasteca Region, Mexico, 2013

**Published:** 2014-06-27

**Authors:** Alberto Díaz-Quiñonez, Irma Hernández-Monroy, Norma Montes-Colima, Asunción Moreno-Pérez, Adriana Galicia-Nicolás, Hugo Martínez-Rojano, Concepción Carmona-Ramos, Miroslava Sánchez-Mendoza, José Cruz Rodríguez-Martínez, Lorena Suárez-Idueta, María Eugenia Jiménez-Corona, Cuitláhuac Ruiz-Matus, Pablo Kuri-Morales

**Affiliations:** 1 Instituto de Diagnóstico y Referencia Epidemiológicos “Manuel Martínez Báez”; 2 Laboratorio Estatal de Salud Pública de Hidalgo; 3 Dirección General de Epidemiología; 4 Subsecretaría de Prevención y Promoción de la Salud

On September 2 and 6, 2013, Mexico’s National System of Epidemiological Surveillance identified two cases of cholera in Mexico City. Rectal swab cultures from both patients were confirmed as toxigenic *Vibrio cholerae* serogroup O1, serotype Ogawa, biotype El Tor. Pulsed-field gel electrophoresis and virulence gene amplification (*ctxA*, *ctxB*, *zot,* and *ace*) demonstrated that the strains were identical to one another but different from strains circulating in Mexico previously. The strains were indistinguishable from the strain that has caused outbreaks in Haiti, the Dominican Republic, and Cuba (1,2). The strain was susceptible to doxycycline, had intermediate susceptibility to ampicillin and chloramphenicol, was less than fully susceptible to ciprofloxacin, and was resistant to furazolidone and trimethoprim-sulfamethoxazole. An investigation failed to identify a common source of infection, additional cases, or any epidemiologic link between the cases. Both patients were treated with a single, 300-mg dose of doxycycline, and their symptoms resolved.

On September 12 and 13, four cases of cholera were identified by the Hidalgo Public Health Laboratory among residents of La Huasteca region, located approximately 75 miles (121 km) east of Mexico City and inhabited mainly by Otomi and Nahuatl speakers. During September 19–December 15, 2013, a total of 175 cases of cholera were confirmed in La Huasteca (159 in Hidalgo, 14 in Veracruz, and two in San Luis Potosí). Cases were defined according to World Health Organization (WHO) guidelines (3). A case of cholera was suspected if, in an area where the disease is not known to be present, a patient aged ≥5 years developed severe dehydration or died from acute watery diarrhea (3). All cases were laboratory-confirmed at the Instituto de Diagnóstico y Referencia Epidemiológicos as toxigenic *V. cholerae*, serogroup O1, serotype Ogawa, biotype El Tor, identical to the Mexico City isolates and indistinguishable from the strain circulating in the Caribbean. All of the cases have been reported to WHO by Mexico’s International Health Regulations Focal Point (4).

Among the 175 cases, 86 (49%) were in females, and the median age of patients was 32 years (range = 3 months–83 years). Only 40 (23%) patients required hospitalization, with an average hospital stay of 36 hours. All patients had acute and watery diarrhea, and 46 (26%) passed “rice-water” stool; 63 (36%) had fewer than five bowel movements per 24 hours, 86 (49%) had vomiting, and 30 (17%) had cramps. Some degree of dehydration was noted in 75 (43%) patients; 37 (21%) suffered mild dehydration (<5% loss of body weight), 32 (18%) moderate dehydration (6%–9% loss), and five (3%) severe dehydration (≥10% loss). One patient died, a woman aged 67 years with a history of diabetes and chronic renal failure. The spectrum of disease seen in this outbreak differed from that of outbreaks in the Caribbean; the proportion of infected persons, incidence of dehydration, mortality rate, and numbers of hospitalizations and complications were smaller in La Huasteca than in the Caribbean (5).

Three quarters of patients were residents of areas neighboring El Tecoluco and El Chinguiñoso streams flowing into the Panuco River. *V. cholerae* isolates recovered from both streams were identical to the outbreak strain. Samples obtained from municipal sewers, fish vendors, restaurants, and drinking water sources were tested to identify potential outbreak sources.

In Mexico, during 1991–2001, a total of 45,062 confirmed cases of cholera occurred, with a 1.1% case-fatality rate. Cases of infection by *V. cholerae* serogroup O1 have occurred sporadically since the end of that epidemic; regular, active surveillance allowed the identification of one case in 2010, one in 2011, and two in 2012, all in the northwestern state of Sinaloa. The first two cases were caused by toxigenic *V. cholerae* O1 serotype Inaba, and the other two by toxigenic *V. cholerae* O1 serotype Ogawa. All of the isolate strains were characterized by Instituto de Diagnóstico y Referencia Epidemiológicos and were identical to the strains circulating in Mexico during 1991–2001 (6).

Health professionals at different levels of the health-care system in Mexico are being trained in cholera prevention, treatment, and control. Public awareness campaigns to safeguard food and water quality, including national radio messages on the prevention of diarrhea, are being carried out in Spanish, Nahuatl, and Otomi languages. Health authorities continue to increase epidemiologic capacity at the national level, ensure the availability of adequate medical management, increase sanitation and access to potable water at the community level, and monitor chlorine levels in drinking water. In addition, continuous microbiologic surveillance for cases of *V. cholerae* infection and *V. cholerae* contamination of reservoirs is in place to promptly detect strains with pathogenic potential.

As a result of these actions, the outbreak in La Huasteca, in which samples from 88% of the cases were collected, was controlled within the first 13 weeks. A mobile microbiology laboratory was used in this area to quickly diagnose and treat patients and to interrupt transmission. Ongoing and continuous microbiologic surveillance of area reservoirs and laboratory investigation of all cases of acute diarrhea have not detected any new cases of cholera since December 17, 2013.
